# Focus on Brain Angiotensin III and Aminopeptidase A in the Control of Hypertension

**DOI:** 10.1155/2012/124758

**Published:** 2012-06-26

**Authors:** John W. Wright, Shigehiko Mizutani, Joseph W. Harding

**Affiliations:** ^1^Departments of Psychology and Veterinary and Comparative Anatomy, Pharmacology, and Physiology and Programs in Neuroscience and Biotechnology, Washington State University, P.O. Box 644820, Pullman, WA 99164-4820, USA; ^2^Department of Medical Science of Proteases, Daiya Building Lady's Clinic, Meieki 3-15-1, Nakamura, Nagoya 450-0002, Japan

## Abstract

The classic renin-angiotensin system (RAS) was initially described as a hormone system designed to mediate cardiovascular and body water regulation. The discovery of a brain RAS composed of the necessary functional components (angiotensinogen, peptidases, angiotensins, and specific receptor proteins) independent of the peripheral system significantly expanded the possible physiological and pharmacological functions of this system. This paper first describes the enzymatic pathways resulting in active angiotensin ligands and their interaction with AT_1_, AT_2_, and mas receptor subtypes. Recent evidence points to important contributions by brain angiotensin III (AngIII) and aminopeptidases A (APA) and N (APN) in sustaining hypertension. Next, we discuss current approaches to the treatment of hypertension followed by novel strategies that focus on limiting the binding of AngII and AngIII to the AT_1_ receptor subtype by influencing the activity of APA and APN. We conclude with thoughts concerning future treatment approaches to controlling hypertension and hypotension.

## 1. Introduction

The first physiological insight into blood pressure (BP) regulation was the isolation of kidney renin by Tigerstedt and Bergman in 1897 [1]. This initial work led to a description of renovascular hypertension in animals and humans by Goldblatt and colleagues. [2]. In 1940, Braun-Menendez and coworkers [3] isolated a vasoconstrictive substance from renal venous blood taken from a Goldblatt hypertensive dog. In this same year, Page and Helmer [4] isolated a “renin activator” after injecting renin into an intact animal. This renin activator was later identified as angiotensinogen. The pressor substance was termed “angiotonin” (also referred to as “hypertension”), and was eventually shown to be an octapeptide [5–7]. It was agreed by Braun-Mendez and Page in 1958 to name this octapeptide “angiotensin.” Since that time extensive physiological, biochemical and behavioral studies have established a prominent role for angiotensin in blood pressure and body water/electrolyte balance.

This paper initially describes the presently identified angiotensin ligands of the renin-angiotensin system (RAS) and details the enzymes involved in their formation and degradation. The two prominent angiotensin receptor subtypes that bind these ligands (AT_1_ and AT_2_) have been characterized, as have the roles of angiotensin II (AngII) and angiotensin III (AngIII) in blood pressure regulation. We next focus on current and novel approaches designed to treat hypertension by manipulating aminopeptidases. We conclude with some thoughts on future directions concerning treatment strategies to control hypertension.

## 2. Formation of Angiotensin Ligands

**Figure 1:**
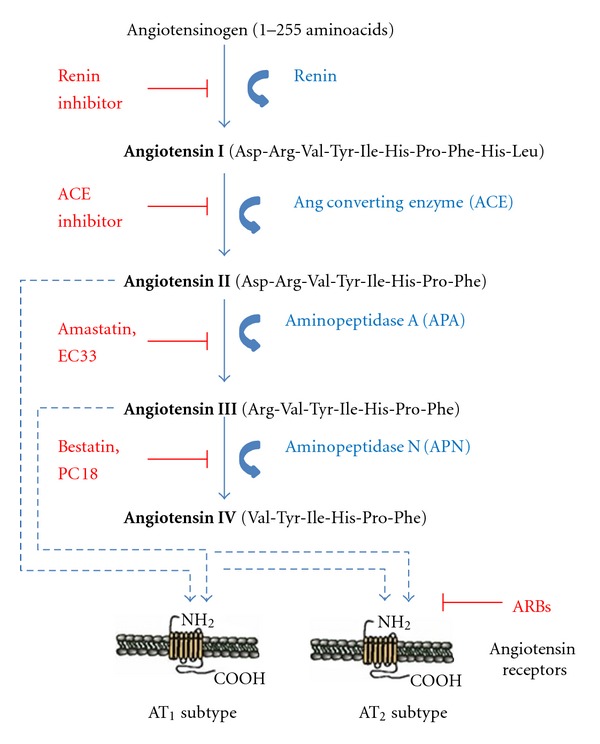
The renin-angiotensin pathway including active ligands (bold), enzymes, receptors, and inhibitors involved in central angiotensin mediated blood pressure. Abbreviations: ACE: angiotensin converting enzyme; APA: aminopeptidase A; APN: aminopeptidase N; ARBs: angiotensin receptor blockers.

Angiotensin peptides are derived from the precursor protein angiotensinogen through several enzymatic conversion pathways (Figure 1 [8–10]). The decapeptide angiotensin I (AngI) is formed by renin (EC 3.4.23.15) acting upon the amino terminal of angiotensinogen [11]. AngI serves as a substrate for angiotensin converting enzyme (ACE: EC 3.4.15.1), a zinc metalloprotease that hydrolyzes the carboxy terminal dipeptide His-Leu to form the octapeptide AngII [8, 12]. This conversion can also be accomplished by the chymotrypsin-like serine protease, chymase [13]. AngII is converted to the heptapeptide AngIII by glutamyl aminopeptidase A (APA: EC 3.4.11.7, or A-like activity) that cleaves the Asp residue at the N-terminal [14–17]. Membrane alanyl aminopeptidase N (APN: EC 3.4.11.2) cleaves Arg at the N-terminal of AngIII to form the hexapeptide angiotensin IV (AngIV). AngIV can be further converted to Ang(3-7) by carboxypeptidase P (Carb-P) and propyl oligopeptidase (PO) cleavage of the Pro-Phe bond. Endopeptidases such as chymotrypsin are capable of cleaving the Val, Tyr, and Ile residues, along with dipeptidyl carboxypeptidase that cleaves the His-Pro bond, reducing AngIV and Ang(3-7) to inactive peptide fragments and aminoacid constituents [8, 18–22].

Some years ago the nomenclature committee of the International Union of Biochemistry [23] indicated that APA was likely identical with APN. However, it has been shown that APA cleaves the N-terminal Asp from AngII, but it also cleaves Arg and Val [24]. The speed of Arg and Val cleavage was facilitated when a combination of APA and placental leucine aminopeptidase (P-LAP) was used [25, 26].

AngII can also be converted to Ang(1-7) by Carb-P cleavage of Phe [27], by the monopeptidase ACE_2_ [28, 29], or by ACE cleavage of the dipeptide Phe-His from Ang(1-9) [30]. Ang(1-7) is further converted to Ang(2-7) by APA acting at the Asp-Arg bond [31]. AngII and AngIII are full agonists at the AT_1_ and AT_2_ receptor subtypes (see [32, 33] for review). AngIV binds with low affinity at the AT_1_ and AT_2_ receptor subtypes, but with high affinity and specificity at the AT_4_ receptor subtype [34–39]. AngI is biologically inactive; while its metabolites AngII and AngIII mediate pressor and dipsogenic effects via the AT_1_ and AT_2_ receptor subtypes [32]. AngIV exerts a much reduced pressor response, also by acting as an agonist at the AT_1_ receptor subtype [40–44].

A counter regulatory axis to the AngII-AngIII/AT_1_ receptor system has recently been proposed consisting of ACE_2_, and Ang(1-7) acting at the Mas receptor. This system appears to act in opposition by promoting vasodilation, antifibrosis, antihypertrophic and antiproliferative effects (see [45, 46] for review) and is further discussed in the next section.

## 3. Characterization of the AT_1_, AT_2_, ****and Mas Receptors

The AT_1_ receptor subtype is a G-protein-coupled receptor with signaling via phospholipase-C and calcium. Thus, the angiotensin ligand binds to the AT_1_ receptor and induces a conformational change in the receptor protein that activates G proteins, which in turn, mediate signal transduction. This transduction involves several plasma membrane mechanisms including phospholipase-C, -A_2_, and -D-adenylate cyclase, plus L-type and T-type voltage sensitive calcium channels [32, 47, 48]. The AT_1_ receptor (now designated AT_1A_) is also coupled to intracellular signaling cascades that regulate gene transcription and the expression of proteins that mediate cellular proliferation and growth in many target tissues. Expression cloning was used to isolate the cDNAs encoding this receptor protein [49, 50], and it was found to be a 7-transmembrane domain protein consisting of 359 aminoacids with a mass of approximately 41 kDa [51]. Subsequently, a second AT_1_ subtype was discovered and designated AT_1B_ that was also cloned in the rat [52, 53], mouse [54], and human [55]. This subtype is approximately 92–95% homologous with the aminoacid sequence of the AT_1A_ subtype [56, 57]. Of these two isoforms, the AT_1A_ subtype appears to be responsible for the classic functions associated with the brain angiotensin system (see [58, 59] for review).

The AT_2_ receptor subtype has been cloned and sequenced using a rat fetus expression library [60, 61], and also evidences a 7-transmembrane domain characteristic of G-protein-coupled receptors; however, it shows only about 32–34% aminoacid sequence identity with the rat AT_1_ receptor. The AT_2_ receptor protein includes a 363 aminoacid sequence (40 kDa) with 99% sequence agreement between rat and mouse and 72% homology with human [32]. Even though this AT_2_ receptor possesses structural features in common with members of the 7-transmembrane family of receptors, it displays few if any functional similarities with this group, although it does appear to be G-protein-coupled [32, 60–62]. While the AT_1_ receptor subtype is maximally sensitive to AngII, it is also responsive to AngIII. The AT_2_ receptor subtype appears to be maximally sensitive to AngIII, but AngII also serves as a ligand at this receptor subtype.

The Mas receptor was discovered in 1986 and was initially categorized as a protooncogene because it transformed NIH 3T3 cells in a tumorigenicity assay (nude mice) using DNA taken from a human epidermoid carcinoma cell line [63]. Subsequently, it was reported that overexpression of Mas in cones of the retina caused cell death but no tumor formation [64]. Further, transgenic mice overexpressing Mas in the brain failed to reveal tumor formation [65]. Young and colleagues [66] measured a high expression of the Mas receptor in the hippocampus and neocortex of the rat brain. The human Mas cDNA sequence indicated an open reading frame coding for a 325 aminoacid protein [63] belonging to the classification of G-protein coupled receptors. Cellular localization of Mas mRNA has been reported using *in situ* hybridization with high levels detected in the rat and mouse hippocampus and piriform cortex [67]. This receptor binds Ang(1-7) with high specificity that could not be displaced using AngII or AngIV [68]. While AngII, and to a lesser extent AngIII, have been considered the active peptides derived from angiotensinogen, convincing evidence exists indicating that Ang(1-7) plays an important role in cardiovascular and pulmonary biology (see [69, 70] for review). Ang(1-7), working through the Mas receptor, initiates many biological responses that are opposite to those attributed to AngII. These include vasodilatory, antihypertrophic, antihyperplastic, and antifibrotic action on the heart and vasculature [71–73]. Unlike AngII, Ang(1-7) is generally synthesized from AngII or Ang(1-9), and not AngI [74]. This synthesis is primarily carried out by ACE_2_ in the heart, vasculature, lungs, and brain [75, 76]. The potential clinically beneficial effects of augmenting the ACE_2_/Ang(1-7)/Mas signaling system has spurred the development of ACE_2_/Ang(1-7)/Mas system potentiating drugs including Mas agonists and ACE_2_ activators [77].

The functions associated with the activation of each of these receptors are presented in Table 1.

## 4. The Pivotal Role of Aminopeptidases in Blood Pressure Regulation

As indicated above, APA hydrolyzes N-terminal acidic aminoacids. It appears that glutamyl derivatives are more effectively hydrolyzed than aspartyl derivatives, thus this enzyme has also been designated glutamyl aminopeptidase (Glu-AP [22]). Aminopeptidase N acts on N-terminal neutral aminoacids, and since alanine is most efficiently cleaved by APN, this enzyme is also known as alanyl aminopeptidase (Ala-AP). In addition, it has been referred to as aminopeptidase M (APM) because of its association with pig kidney microsomal membrane fraction from which it was originally purified. Aminopeptidase B (AP-B; EC 3.4.11.6) hydrolyzes basic aminoacids at the N-terminal and is also known as arginine aminopeptidase (Arg-AP). Cystinyl aminopeptidase (Cys-AP; EC 3.4.11.3) hydrolyzes the N-terminal cysteine next to tyrosine of oxytocin and vasopressin. Thus, it is frequently referred to as oxytocinase or vasopressinase. P-LAP appears to be equivalent with Cys-AP; while insulin-regulated aminopeptidase (IRAP) is the rat homologue of Cys-AP [78, 79]. Since these enzymes are involved in metabolizing angiotensins, they have often been grouped under the name “angiotensinases.”

## 5. The Emergence of AngIII and APA as Key Factors in Sustaining Hypertension

It is now reasonably well established that a major reason for high BP in the spontaneously hypertensive rat (SHR) model is an overactive brain RAS, and the primary ligand in this regard may be AngIII. Originally, it was reported that intracerebroventricular (i.c.v.) infusion of AngII or AngIII produced dose-dependent elevations in BP with the magnitude of these elevations reasonably equivalent [80, 81]. Higher rates of neuronal firing were shown with the microiontophoresis of AngIII, as compared with AngII, in both the subfornical organ (SFO) [82] and paraventricular nucleus (PVN) [83, 84], two brain structures responsible for mediating BP. Harding et al. [85], using a push-pull cannula into the PVN, showed that 93% of the releasable angiotensin coeluted with AngIII; while only 7% eluted with AngII. This suggested that the conversion of AngII to AngIII is rapid and a critical step in maintaining BP. It was next discovered that SHRs were more sensitive to i.c.v.-infused angiotensins than normotensive Wistar-Kyoto (WKY) and Sprague-Dawley rats. Further, these angiotensin-induced elevations in BP remained high for longer durations in the SHR. Pretreatment with the APN inhibitor bestatin potentiated and prolonged the pressor response to both AngII and AngIII. These results suggested that inhibiting the conversion of AngIII to the hexapeptide AngIV prolonged the half-life of AngIII, thus providing a potential mechanism for the higher BP seen in the SHR, that is, a delay in the conversion of AngII to AngIII, and Ang III to AngIV. The extended half-life of brain AngII and AngIII would be expected to prolong heightened activation of the AT_1_ receptor subtype and thus maintain an elevated BP. Pretreatment with bestatin potentiated and prolonged the pressor response to both AngII and AngIII. We interpreted these results to suggest that inhibiting the conversion of AngIII to the hexapeptide AngIV prolonged the half-life of AngIII, thus providing a plausible explanation for the higher sustained blood pressure seen in the SHR. Next, we demonstrated that the i.c.v. infusion of bestatin elevated BP in both SHR and WKY rats [86]; while the infusion of Ala-AP significantly reduced BP in SHR and WKY rats. Pretreatment with the nonselective angiotensin receptor antagonist, [Sar^1^, Thr^8^] AngII (sarthran, predecessor to Losartan) significantly diminished this Ala-AP-induced drop in BP in SHRs and promoted recovery of BP and heart rate to baseline values in members of both strains. Taken together, these results pointed to an important role for brain AngIII interacting with the AT_1_ receptor subtype.

We suspected a disruption in brain aminopeptidase activity in the SHR [87, 88]. To test this hypothesis replacement Glu-AP was i.c.v.-infused in order to promote the hydrolysis of AngII to AngIII. Results indicated that BP was elevated in both SHR and WKY rats [89]. In contrast, the i.c.v. infusion of Ala-AP, which hydrolyzed AngIII, significantly decreased BP in WKY rats, but especially in SHRs. By the use of metabolically resistant analogues of AngII ([D-Asp^1^] AngII) and AngIII ([D-Arg^1^] AngIII), it was shown that the AngIII analogue generated a much greater pressor response in the SHR than the AngII analogue when i.c.v.-infused [90]. Taken together, these results supported the notion that interfering with the conversion of AngII to AngIII results in a significant reduction in BP [91], and brain AngIII is a much more potent pressor agent than AngII.

## 6. Current Treatment Strategies

In the periphery, the cardiovascular effects due to excessive formation of AngII bear a remarkable resemblance to those observed in response to overactivation of the sympathetic component of the autonomic nervous system and excess norepinephrine. These include ventricular hypertrophy, vasoconstriction, and sodium retention. The synthesis cascade involved in the formation of AngII offers several opportunities for clinical intervention, as does the interaction of AngII and AngIII with the AT_1_ receptor subtype. These potential points of intervention include treatment with renin inhibitors, ACE inhibitors, APA inhibitors, aldosterone inhibitors, and angiotensin receptor blockers (ARBs).

### 6.1. Renin Inhibitors

As cardiovascular deficiency progresses, decreased renal perfusion occurs accompanied by increased release of norepinephrine resulting in increased juxtaglomerular renin release into the circulation. Renin is an aspartic proteinase designed to cleave the Leu-Val bond at the amino terminal of angiotensinogen, thus forming the decapeptide AngI. This conversion is considered to be the first rate-limiting step of the RAS. First- and second-generation renin inhibitors were peptidomimetics effective at lowering BP in hypertensive patients; however, these inhibitors exhibited very poor oral bioavailability and were subject to high production costs. Third-generation renin inhibitors are nonpeptides, such as Aliskiren, and appear to be more therapeutically promising in that they evidence good duration of action and bioavailability [92].

### 6.2. ACE Inhibitors

The next potential point of intervention in the angiotensin synthetic cascade is the conversion of AngI to AngII, where ACE inhibitors have utility. ACE hydrolyzes the carboxy terminal dipeptide His-Leu to form AngII [8]. ACE inhibitors have become a primary therapeutic treatment for hypertension and systolic heart failure. The myocardium of patients with failing hearts shows increased levels of ACE mRNA, ACE protein, and ACE activity [93]. The notion that hemodynamic load determines the level of RAS expression in the heart is supported by the observation that ACE increases as the severity of heart failure increases. In agreement, left ventricle ACE and AngII levels change with diastolic ventricular wall stress [94]. A disadvantage of ACE inhibitors concerns their ability to prevent the breakdown of bradykinin to inactive peptide fragments. This can result in an accumulation of bradykinin that contributes to angioedema and cough. In some patients, these side effects can be sufficiently troublesome to result in discontinuation of the medication. Even so ACE inhibitors appear to provide long-term positive effects in many patients with heart failure. Paradoxically, these beneficial effects may be due to the buildup of bradykinin which facilitates reduced norepinephrine synthesis and release, vasodilation, and general antiproliferative effects [95].

### 6.3. Aldosterone Receptor Antagonists

Early on, it was assumed that ACE inhibitors would adequately limit the synthesis of aldosterone given AngII's ability to facilitate aldosterone production. However, ACE inhibition causes a transient depression in circulating aldosterone levels followed by recovery after only a few days. This turns out to be a particularly important limitation of ACE inhibition in the treatment of heart failure [96]. Elevations in circulating aldosterone have long been known to increase sodium reabsorption and potassium excretion, but increased aldosterone elevates cytokines, promotes vascular inflammation and endothelial malformation, and also myocardial fibrosis and hypertrophy. Animal model work has demonstrated a significant role for elevated aldosterone levels contributing to myocardial fibrosis [97] and left ventricular remodeling [98]. McKelvie and colleagues [99] showed that standard therapy (ACE inhibitor, ARBs, and *β*-adrenergic receptor antagonist) failed to reduce circulating aldosterone levels in patients with heart failure due to left ventricular systolic dysfunction. Pitt et al. [100] treated hypertensive patients, complicated by left ventricular hypertrophy, with the selective aldosterone receptor antagonist Eplerenone, the ACE inhibitor Enalapril, or a combination of the two. Eplerenone (mean = −14.5 g) decreased left ventricular mass similar to Enalapril (−19.7 g), but combined treatment (−27.2 g) was more effective. These results suggest that an ACE inhibitor alone is not sufficient to maximize left ventricular mass reduction. 

### 6.4. Angiotensin Receptor Blockers

Given the importance of the AT_1_ receptor subtype's role in contributing to the cardiovascular continuum, it seems reasonable that specific angiotensin receptor blockers could significantly reduce cardiovascular risk factors. Initial studies in canine models of left ventricular diastolic hypertrophy revealed beneficial effects due to ARB treatment [101]. The Losartan Intervention for Endpoint Reduction in Hypertension (LIFE) study found regression of left ventricular hypertrophy following long-term treatment with Losartan or Atenolol [102]. A study by Díez and colleagues [103] treated hypertensive patients with severe or nonsevere myocardial fibrosis with Losartan for 12 months. These patients demonstrated a clear improvement in left ventricular chamber stiffness as correlated with a reduction in collagen fraction volume, particularly in the severe fibrosis patients.

The Candesartan in Heart Failure Assessment of Reduction in Mortality and Morbidity (CHARM) study evaluated the contribution of ARBs in combination with ACE inhibitors, *β*-adrenergic antagonists, and aldosterone receptor antagonists in a large group of heart failure patients possessing left ventricular ejection fraction greater than 40%. There was a trend toward fewer cardiovascular problems with Candesartan treatment than placebo [104]. However, there was an accompanying hyperkalemia and/or elevated creatinine levels in these patients.

## 7. Novel Treatment Strategies

Three novel approaches to the treatment of hypertension have focused on inhibiting the enzymes involved in the formation of AngII and AngIII, or incrementing the enzymatic pathway such that these angiotensins are rapidly degraded.

### 7.1. Reduction in Brain AngIII Formation via Inhibition of APA

The Llorens-Cortes research group has extended initial work completed with amastatin and bestatin by designing inhibitors with improved specificity. Reaux et al. [105] synthesized two inhibitors: one specific for APA referred to as EC33 and a second specific to APN called PC18 (m.w. = 120–130 kDa for the monomer). Intracerebroventricular pretreatment with EC33 blocked the elevation in BP typically induced by the i.c.v. infusion of AngII in anesthetized SHRs. These results suggested that the AngII pressor response depended upon conversion of AngII to AngIII. Further, the i.c.v. infusion of EC33 caused a large dose-dependent decline in BP in conscious SHRs, as well as conscious DOCA-salt treated rats (a salt/volume-dependent and renin-independent model of hypertension) (Table 2 [106, 107]). This effect could not be induced by intravenous infusion of EC33, suggesting that EC33 did not penetrate the BBB and/or was not peripherally effective. These results were interpreted to suggest that in addition to reducing conversion of AngII to AngIII, EC33 also encouraged degradation of accumulating endogenous AngII by peptidases including endopeptidases and carboxypeptidases, for example, ACE_2_ [108–110]. This would be expected to result in the formation of smaller AngII fragments possessing very low, or no, AT_1_ receptor affinity.

The conclusion that AngIII is the active ligand acting at the AT_1_ receptor subtype was further supported in that i.c.v.-infused PC18 in SHRs significantly increased BP [105]. This PC18-induced pressor response could be prevented by pretreatment with the specific AT_1_ receptor antagonist Losartan, but not by the specific AT_2_ receptor antagonist PD123319. Thus, these results suggest that the inhibition of APN results in the accumulation of endogenous AngIII with increased binding at the AT_1_ receptor subtype causing an increase in BP. Our laboratory confirmed these basic findings using metabolic-resistant analogues: D-Asp^1^-AngII and D-Arg^1^-AngIII i.c.v.-infused in conscious normotensive rats in the presence or absence of EC33 or PC18 (Table 2 [91]). And finally the i.c.v. infusion of APA significantly increased blood pressure in conscious SHRs; while the i.c.v. infusion of APN decreased BP [88]. This hypertensive effect was attributed to increased endogenous levels of AngIII, and the hypotensive effect to increased AngIII degradation. Along these lines, Song and colleagues [116] reported that i.c.v. infusion of an antiserum against APA activity significantly reduced AngII-induced BP increases by *∼*60%. Taken together, these findings illustrate the importance of AngII conversion to AngIII in the brain but not in the periphery, regarding the mediation of BP. Thus, endogenous brain AngIII appears to exert a maintained tonic influence on centrally controlled BP in conscious normotensive and hypertensive animals. It is noteworthy that the inhibition of APA reduced BP to normotensive levels in hypertensive rat models.

These findings have resulted in the development of a selective orally active APA inhibitor (RB150) capable of crossing the blood-brain barrier and functioning as a antihypertensive drug (Table 2 [107]). RB150 is a pro-drug of EC33 formed from two molecules of EC33 linked by a disulfide bridge. The presence of the thiol group in the disulfide bridge prevents interaction with the zinc atom at the APA site. This bridge appears to permit BBB penetration, and once in the brain the disulfide bridge is removed by reductases, it yields two active EC33 molecules [117]. RB150 has been shown to function as an antihypertensive drug when given intravenously [107] or by gavage [114].

### 7.2. Facilitation of Angiotensin Degradation

There is considerable discrepancy in findings regarding the importance of AngIII as a mediator of peripheral BP. For example, Ahmad and Ward [118], using amastatin and bestatin, failed to confirm an important role of APA in the systemic RAS. In contrast to the finding that i.c.v.-infused EC33-induced a nearly complete inhibition of APA in the rat brain [108], intravenous infusion of EC33 resulted in only limited blockade of plasma AngII conversion to AngIII [117]. There are several possible explanations for these differences comparing the brain and the periphery: (1) AngIII could be more rapidly metabolized than AngII in the periphery. However, our laboratory has determined the half-life of AngIII in the vasculature of normotensive rats to be 16.3 seconds; while the half-life of AngII was 12.5 sec [119]; (2) the major enzyme responsible for converting AngII to AngIII in the periphery is not APA. This is unlikely given the number of studies pointing to an important role for APA in this regard (see [16–18, 20, 22, 105, 120–123] for review); (3) related to the above, alternative enzymatic pathways could be present in the peripheral circulation, for example, ACE_2_ may play an important role [29, 69, 124]. Clearly, there are many more enzymes involved in the metabolism of angiotensin than APA and APN [10].

As described above, the removal of the C-terminal His-Leu from AngI by ACE to form AngII, the conversion of AngII to AngIII due to the removal of Asp by APA, and the subsequent conversion of AngIII to AngIV with the removal of Arg by APN, are critical steps in determining the availability and survival rates of circulating AngII and AngIII. The hydrolysis of AngII to AngIII by APA could be the rate-limiting step in determining the level of activity of AT_1_ receptor activation in the periphery (see [25, 125] for review). In agreement with the importance of APA, recombinant human APA [126] has been shown to have value as an antihypertensive treatment in SHRs [127]. The effective dose of APA was approximately one-tenth that of the ARB Candesartan. This approach was spawned from the original observation that the SHR resembles human essential hypertension in that ACE inhibition, or ARB treatment, normalized BP [128, 129]. These results emphasize the importance of the RAS to the hypertension seen in this animal model. It was next confirmed that intravenous infusion of APA plus APN (purified from human placenta) lowered AngII-induced hypertension in normotensive rats [130]; while APA normalized BP in SHRs [131]. These results suggested that elevated APA could be effective in controlling high BP; while impairment of APA activity contributed to the development of hypertension. This observation supports the finding that SHRs have impaired aminopeptidase activity [132]; while renal APA activity in the SHR was found to be lower than in age-matched WKY rats [133].

The use of purified and recombinant APA is certainly worth considering for the treatment of hypertensive emergencies such as acute hypertensive crisis, preeclampsia, acute heart failure, and hypertensive encephalopathy [134, 135]. However, the notion that AngIII is the primary ligand acting at the AT_1_ receptor subtype in the brain's control of BP points to the importance of inhibiting APA in hypertension (Llorens-Cortes hypothesis). On the other hand, the proposal that AngII is the primary ligand acting at the AT_1_ receptor subtype suggests that elevations in APA should be efficacious in controlling hypertension (Mizutani hypothesis). Additional research effort will be necessary to resolve this important issue.

### 7.3. APN Inhibitors

Finally, there is growing interest in the development of APN inhibitors [136]. The human APN gene consists of 20 exons and maps to chromosome 15q25-26 [137]. With the use of adipocyte-derived APN, 33 polymorphisms have been identified [138]. Two of eight missense polymorphisms have been associated with arterial blood pressure, and one (Lys528Arg) has been linked to essential hypertension [138, 139]. This Lys528rg polymorphism has been reported to inhibit APN enzymatic activity [126]. Increased urinary APN levels have been measured in renal transplant rejection patients [140] and renal cell carcinoma patients [141]. A recent study by Padia and colleagues [142] noted a defect in sodium excretion in young SHRs prior to the onset of hypertension. In this regard, young WKY rats evidenced twice the excretion rate of sodium attributed to reduced renal proximal tubule sodium reabsorption. The renal interstitial infusion of PC18 corrected this natriuresis by incrementing sodium excretion. Thus, the inhibition of AngIII metabolism by inhibiting APN corrected this proximal tubule defect in sodium excretion in SHRs. The renal interstitial infusion of AngIII produced a natriuresis in normotensive rats that was dependent on the AT_2_ receptor subtype [143]. The authors argue that conversion of AngII to AngIII is necessary for this natriuresis, and AT_1_ receptor blockade with Candesartan failed to impact these AngIII-induced effects. The potential use of APN inhibitors for inflammatory disease and malignancies is being considered [144]; however, the testing of APN inhibitors to treat hypertensive patients is just now beginning. It should be noted that the use of APN inhibitors to control hypertensive disorders is controversial, and this area is in need of additional careful research attention.

## 8. Conclusion

Research interest in understanding the role of the RAS in the control of BP has been ongoing for over 100 years, and yet new discoveries continue to be made. Hypertension is present in approximately 16% of the adult population of the world, and 95% are categorized as essential hypertension treated with the major classes of antihypertensives including diuretics, ACE inhibitors, ARBs, *β*-blockers, and calcium channel blockers [145, 146]. Despite the availaiblity of over 75 antihypertensive drugs, approximately 65% of hypertensive patients are not able to control their elevated BP [147]. And the incidence of “resistant hypertension” despite the combination of three or more drugs is around 15% of the hypertensive population [147]. Thus, we are in need for novel approaches to better treat these patients. It now appears that the importance of brain AngIII has been significantly underestimated in that the conversion of brain AngII to AngIII by APA has a major influence upon the regulation of BP and release of vasopressin. In this regard, AngIII may exert a persistent tonic influence upon BP. It is surprising that a selective APA inhibitor such as EC33 decreases BP when given centrally but not peripherally. It must be added that available results concerning RB150 are consistent in this regard. With respect to the potential use of an orally active APA inhibitor, a number of important issues remain including but not limited to: (1) the relative importance of AngIII and APA to blood pressure control comparing the brain and peripheral systems; (2) the influence of other peptidases upon the formation of angiotensin ligands; (3) the significance of the AT_2_ receptor subtype, and Ang(1-7) acting at the Mas receptor, in the control and treatment of essential hypertension.

## Figures and Tables

**Table 1:** Functions associated with ligand activation of the AT_1_, AT_2_, and Mas receptors.

AT_1_ receptor subtype
Vasoconstriction
Aldosterone release
Vasopressin release
Cardiac hypertrophy
Fibrosis
Proliferation
Inflammation
Platelet aggregation
Oxidative stress
Endothelial disruption

AT_2_ receptor subtype
Vasodilation
Antifibrotic
Antiproliferative
Antihypertrophic
Antithrombotic

Mas receptor
Vasodilation
Antifibrotic
Antihypertrophic
Antithrombotic
Promotes endothelial function

**Table 2:** Summary of investigations supporting a role for EC33 and PC18 (EC27) in blocking the release of vasopressin and controlling hypertension in animal models.

Preparation	Finding	Reference
Synthesis of EC33	Designed by Chauvel as a specific inhibitor of APA	[111]
	Synthesized by Reaux and colleagues	
i.c.v. infusion-mice	EC33 increased the half-life of [^3^H]AngII by 2.6 fold and blocked the formation of [^3^H]AngIII	[108]
	PC18 and EC27 increased the half-life of [^3^H]AngIII by 3.9 and 2.3 fold, respectively	[108, 111]
	EC33 reduced AngII-induced vasopressin release in a dose-response-dependent fashion	[111]
	Injection of PC18 and EC27 increased vasopressin release	[111]
Normotensive rats	Recorded from vasopressinergic neurons in the SON	
(urethane-anesth.)	i.c.v. infusion of AngII and AngIII significantly increased firing rate. i.c.v. infusion of EC33 abruptly stopped firing rate for 4–6 min. i.c.v. infusion of AngII followed by EC33 prevented the increase in firing rate to AngII	[112]
i.c.v. infusion-SHRs	EC33 blocked AngII-induced pressor responses	[105, 113]
i.c.v. infusion-normotensive rats	EC33 blocked the pressor response induced by AngII and D-Asp^1^AngII but had no effect on the pressor responses induced by AngIII or D-Arg^1^AngIII. PC18 extended the duration of the D-Asp^1^AngII-induced pressor response 2.5 fold, and the duration of the D-Arg^1^ AngIII-induced pressor response by 10 to 15 fold. Pretreatment with Losartan blocked these pressor responses, indicating AT_1_ receptor involvement	[91]
Synthesis of RB150	Designed by Fournie-Zaluski	[107]
DOCA-salt rats	Intravenous administration of RB150 significantly reduced BP for 24 hours	[107]
	Oral administration of RB150 significantly reduced BP for 7 hours	[114, 115]
